# The Association between ATM IVS 22-77 T>C and Cancer Risk: A Meta-Analysis

**DOI:** 10.1371/journal.pone.0029479

**Published:** 2012-01-19

**Authors:** Lin Zhao, Aihua Gu, Guixiang Ji, Peng Zou, Peng Zhao, Ailin Lu

**Affiliations:** 1 Department of Neurosurgery, The First Affiliated Hospital of Nanjing Medical University, Nanjing, China; 2 School of Public Health, Nanjing Medical University, Nanjing, China; 3 Nanjing Institute of Environmental Sciences, Key Laboratory of Pesticide Environmental Assessment and Pollution Control, Ministry of Environmental Protection, Nanjing, China; Institute of Zoology, Chinese Academy of Sciences, China

## Abstract

**Background and Objectives:**

It has become increasingly clear that ATM (ataxia-telangiectasia-mutated) safeguards genome stability, which is a cornerstone of cellular homeostasis, and ATM IVS 22-77 T>C affects the normal activity of ATM proteins. However, the association between the ATM IVS 22-77 T>C genetic variant and cancer risk is controversial. Therefore, we conducted a systematic meta-analysis to estimate the overall cancer risk associated with the polymorphism and to quantify any potential between-study heterogeneity.

**Methods:**

A total of nine studies including 4,470 cases and 4,862 controls were analyzed for ATM IVS 22-77 T>C association with cancer risk in this meta-analysis. Heterogeneity among articles and their publication bias were also tested.

**Results:**

Our results showed that no association reached the level of statistical significance in the overall risk. Interestingly, in the stratified analyses, we observed an inverse relationship in lung and breast cancer.

**Conclusion:**

Further functional research on the ATM mechanism should be performed to explain the inconsistent results in different cancer types.

## Introduction

Cancer is a multi-factorial disease that results from complex interactions between environmental and genetic factors [1]. The genetic factors contribute more to the causation of cancer than do lifestyle or environmental factors. In terms of genetic factors, the road to cancer is paved with alterations in the sequence and organization of the cellular genome that range from single-nucleotide substitutions to gross chromosomal aberrations [2]. In recent years, studies based on the candidate-polymorphism approach markedly increased the number of associations between polymorphism and cancer risk that could be tested.

Ataxia-telangiectasia (A–T) is a rare autosomal recessive disorder that affects many parts of the body and has an exceptionally high incidence of cancer, including breast cancer, leukemia, and lymphoma [3]–[5]. A–T is caused by mutations in the ataxia-telangiectasia- mutated (ATM) gene [6]. The ATM gene is known to be involved in the cellular response to DNA breaks at several levels, including cell cycle checkpoint activation, DNA repair, and induction of apoptosis [7]. The protein encoded by this gene belongs to the PI3/PI4-kinase family. The human ATM gene has been mapped to chromosome 11q22–23, and it spans 150 kb and comprises 66 exons [6].

DNA damage jeopardizes cellular homeostasis and initiates a response that activates various repair mechanisms that recognize specific DNA lesions [8]. Double-strand breaks (DSBs) are among the various types of DNA lesions that are caused by a range of DNA-damaging agents, such as ionizing radiation and reactive oxygen, and these DNA lesions are deadly. ATM gene plays a key role in the recognition, signaling, and repair of DNA DSBs [2], [9]. ATM also responds to damage caused during meiosis and mitosis or by free radicals generated during the metabolism of estrogens or environmental chemicals. In addition, ATM functions as a regulator of a wide variety of downstream proteins, including tumor suppressor P53, BRCA1, oncogenic protein MDM2, checkpoint kinase CHK2, checkpoint protein RAD50 and DNA repair protein NBS1 [2], [7]. Without these functions, cellular mitosis is prone to the replication of damaged DNA templates and the subsequent generation of damaged chromosomes. It is extremely likely that cancer originates from these altered cells. ATM safeguards genome stability which is a cornerstone of cellular homeostasis. After the identification of the ATM gene in 1995 [10], numerous studies have demonstrated that individuals with ATM have a high incidence of malignancies, particularly breast cancer [7], [11], [12].

Polymorphisms in ATM, which affect normal protein activity, may alter the efficiency of cell cycle checkpoint activation, DNA repair and induction of apoptosis and lead to genetic instability and increased cancer risk. A single-nucleotide polymorphism (SNP) IVS 22–77 T>C (rs664677) is located within intron 22 of the ATM gene and has a minor allele frequency higher than 10%. In addition, IVS 22–77 T>C is in tight linkage disequilibrium with IVS48 _ 238 G, another ATM variant allele that was shown to be an association with breast-cancer risk [13]. To date, many molecular epidemiological studies have evaluated the role of ATM IVS 22–77 T>C in cancer development within populations of different ethnicities [13]–[21]. However, although some of the case-control studies have reported an association with the risk of cancer [13]–[16], [20], other studies have failed to demonstrate any association [17]–[19], [21]. Inconsistencies among previous studies might be due to multiple ethnicities, random errors, and moderate sample sizes. Therefore, the aim of this study was use a meta-analysis approach to evaluate whether the ATM rs664677 polymorphism is actually associated with disease risk.

## Materials and Methods

### Eligible studies

For the literature review, we searched the PubMed and Embase databases (the last search was conducted on May 31, 2011) using the following search terms: “ATM” and “cancer” or “tumor” and “polymorphism” or “variant”. In addition, we screened the reference lists for all included studies, reviews and meta-analyses.

### Validity assessment

Previous studies were included if they contained sufficient published data regarding the following information: 1) The ATM rs664677 polymorphism and cancer risk; 2) A human case-control study of a polymorphism associated with cancer risk; and 3) The genotype frequencies for both cancer cases and controls. Primary reasons for the exclusion of studies are listed as follows: 1) the literature did not contain information regarding cancer research; 2) the study duplicated a previous publication; 3) the study reported no usable data; and 4) the study only involved a case population.

### Data extraction

The data extracted from each eligible publication included the following information: the first author's name, the year published, the year the data were collected, the country in which the study was conducted, the ethnicities of the individuals involved, the cancer type, the source of the controls used, the matching criteria, the genotyping method, the specimen type, the sample size, the genotypic frequencies for experimental cases and controls, and the Hardy-Weinberg equilibrium (HWE) among the controls. Specifically, each selected case was classified as population-based, hospital-based, or mixed. The ethnicity was classified as Asian or European. If the ethnicity was not reported, we considered the ethnicity of the source population of the country where the study was performed.

### Study characteristics

The data from nine cancer case-control publications was used in these analyses. A summary of the individual studies is given in Table 1. Because one study presented the data for genotypes as ‘CC and CT/TT’ without presenting data for all three genotypes, we calculated the odds ratio (OR) for recessive models by statistical analysis [21].

**Table 1:** Overview of the 9 studies included in the pooled reanalysis with individual data.

First author	Year	year of data collection	Country	Ethnicity	Cancer type	Source of controls	Matching criteria	Genotyping method	Cases/controls	HWE
Lo [14]	2010	2002.1–2006.12	China	Asian	Lung	PCC	age, gender, and smoking status	MassARRAY	730/730	0.41
Lee [15]	2010	2001–2003	Korea	Asian	Breast	HCC	age	PCR, TaqMan	206/253	0.49
Akulevich^(a)^ [16]	2009	—	Japan	European	Papillary thyroid carcinoma	PCC	age, IR-exposed status	PCR-RFLP	88/133	0.31
Akulevich^(b)^ [16]	2009	—	Japan	European	Papillary thyroid carcinoma	PCC	age, Non-exposed	PCR-RFLP	87/398	**0.00** *
Li [17]	2009	2000–2007	USA	European	Pancreatic Cancer	PCC	age,sex, race.	TaqMan	734/780	0.44
Yang [21]	2007	1995–2007	USA	European	Lung	PCC	Age, gender, ethnicity and smoking status	TaqMan	547/540	0.34
Landi [18]	2006	1998.2–2002.10	Romania,Hungary,Poland,Russia,Slovakia,Czech Republic	European	Lung	MIXED	random	PCR, microarray	299/317	0.05
Kim [19]	2006	2001–2003	Korea	Asian	Lung	HCC	Age, sex,family history of cancer	PCR, SNPstream	616/616	0.06
Lee [20]	2005	1995–2003	Korea	Asian	Breast	MIXED	reproductive/parity factors	PCR, TaqMan	996/1181	0.17
Angele [13]	2003	1996.2–2002.4	France	European	Breast	PCC	age	PCR-RFLP	254/312	0.87

HCC, hospital-based case–control; PCC, population-based case–control; PCR, polymerase chain reaction; RFLP, restriction fragment length polymorphism; HWE, Hardy-Weinberg equilibrium.

*Controls in Hardy–Weinberg equilibrium: P<0.05.

### Statistical analysis

All statistical tests performed in this study were two-tailed and p values less than 0.05 were considered significant, unless otherwise stated. Statistical analyses were performed using Stata, version 11.0.

For the control groups of each study, the allelic frequency was calculated, and the observed genotype frequencies of the rs664677 polymorphism were assessed for Hardy-Weinberg equilibrium using the test. The publication by Natallia M Akulevich et al. [16] presented two separate case-control studies. Each study was considered separately for the pooling analysis. Hence, a total of nine publications including ten studies were included in the final meta-analysis. All studies with control groups that were not in HWE (p<0.05) were excluded.

The strength of the association between the rs664677 polymorphism and cancer risk was evaluated by the odds ratios (ORs) with 95% confidence intervals (CIs). Pooled estimates of the ORs and 95% CIs were calculated by logistic regression. The pooled ORs were calculated for the heterozygote comparison (CT versus TT), homozygote comparison (CC versus TT), dominant model (CT/CC versus TT) and recessive model (CC versus CT/TT), respectively. The values for the ORs and CIs for each individual were considered twice. The meta-analyses were stratified by cancer type, ethnicity and source of the controls if the data permitted; a minimum of three data sources were required. Of the individual studies included in this pooled analysis, only one source of controls per study was found in all but two cases; these studies were combined into the “mixed” group.

The evaluation of the meta-analysis results included an examination of the heterogeneity, an analysis of the sensitivity, and an examination for bias. The chi-squared-based Q-statistic test was used to assess the between-study heterogeneity, and it was considered significant if P<0.10. The fixed and random effects models were performed, respectively, to combine values from each of the studies based on the Mantel-Haenszel [22] and the DerSimonian and Laird [23]methods. When the effects were assumed to be homogenous, the fixed-effects model was used; otherwise, it was more appropriate to use the random-effects model. The sensitivity analyses were performed to assess robustness and examine the results of our meta-analyses for possible biases.

The inverted funnel plots and Egger's regression test were used to investigate the publication bias. The potential publication bias was assessed with funnel plots of the effect sizes versus the standard errors; the Begg's test was used to identify the significant asymmetry. An asymmetric plot suggests possible publication bias. The bias due to results from small studies was evaluated by the modified Egger's test, which corrected for potential type I errors [24].

## Results

### Flow of included studies

**Figure 1:**
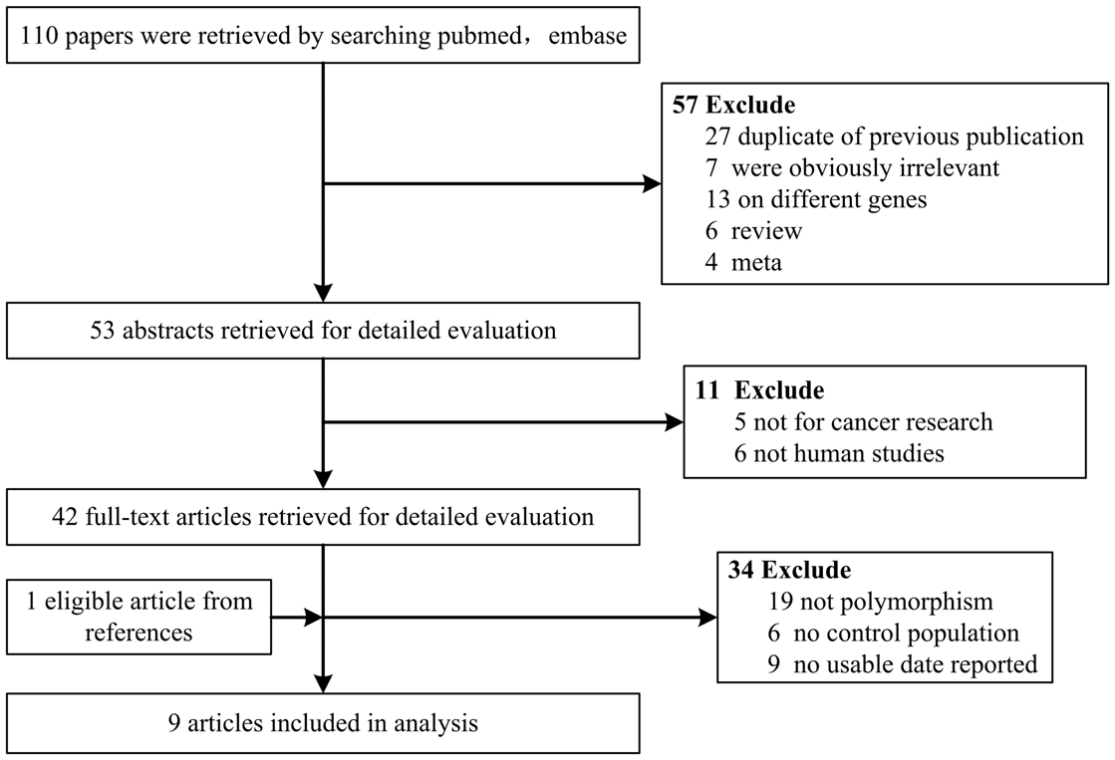
Studies identified with criteria for inclusion and exclusion.

A total of 110 publications were relevant to the search words. Seven studies were obviously irrelevant. Forty studies were excluded because they were duplicates of previous publications (27 studies) or on different genes (13 articles). Four of the articles were meta-analyses, and six of the publications were reviews. Among the remaining 53 publications, six of the articles were not human studies, five of the publications were not for cancer research and six articles had no control population. Another twenty-eight studies were also excluded because they did not present detailed genotyping information (19 articles) or did not report usable data (9 articles). Finally, the references from all included studies reviews and meta-analyses were screened. One additional eligible article was retrieved. Overall, nine studies, involving 4,470 cases and 4,862 controls, concerning ATM rs664677 polymorphism and cancer susceptibility were available for this meta-analysis (Figure 1).

### Characteristics of studies

The studies investigating different cancers, multiple ethnicity or different sources of controls were separated into multiple studies in a subgroup analysis. In addition, one study [21] that only provided the total number of common genotypes (TT and CT) was included in the analysis for the recessive model but not for other genetic models. For the ATM rs664677 polymorphism, there were four studies of Asian descendents and five studies of European descendents. Finally, our meta-analysis consisted of nine case-control studies: three breast cancer studies, four lung cancer studies, one papillary thyroid carcinoma study, and one pancreatic cancer study; in most cases, the cancers were diagnosed histologically or pathologically. In addition, five studies were population-based and two studies were hospital-based; the two publications that did not provide detailed information regarding the source of the controls were mixed. The genotype distributions in the controls for all studies were consistent with Hardy-Weinberg equilibrium, except for a part of one study [16] (Table 1).

### Quantitative synthesis

**Figure 2:**
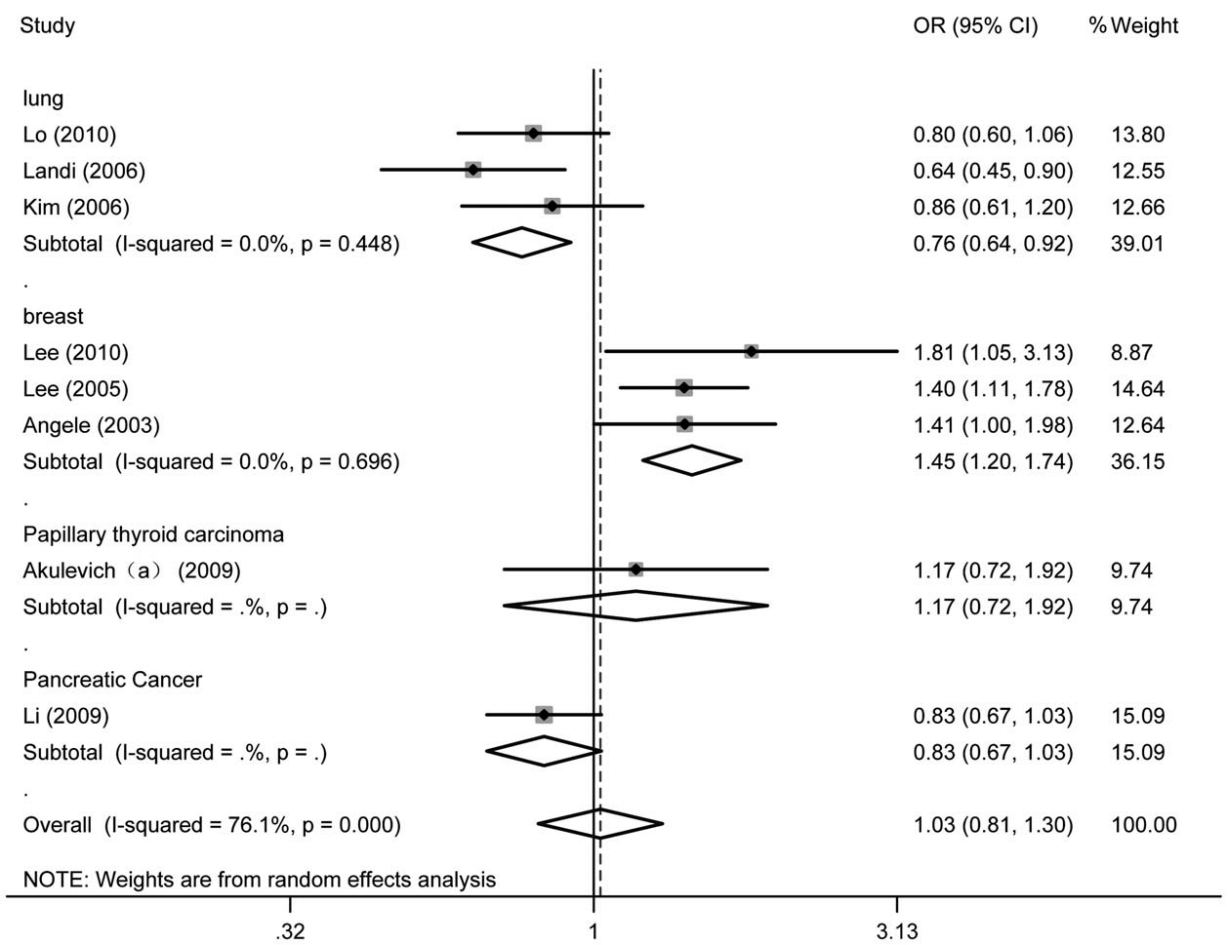
Forest plots of effect estimates for cases and controls of 9 individual studies stratified by type of study (dominant, random-effects model).

It is known that the frequency of the ATM rs664677 polymorphism varies between ethnic groups. For the European populations (n = 1542), the cc allele frequency was 40.9% (95% CI = 33.4–48.4), which was significantly lower than that of the Asian population (n = 2780, 60.6%, 95% CI = 57.5–63.8; Figure 2).

**Figure 3:**
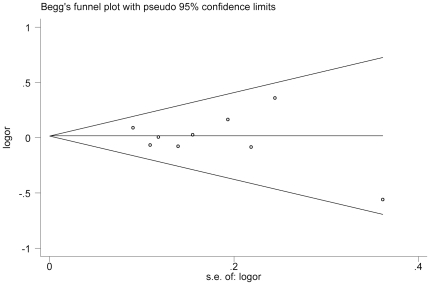
Funnel plot analysis to detect publication bias. Each point represents a separate study for the indicated association (CC VS CT/TT).

The individual risk estimates (Table 2) were calculated and presented as forest plots (Figure 3a) by study type for all nine studies included in the analysis. Overall, no significant association between the ATM rs664677 polymorphism and cancer risk was observed in any genetic model (heterozygote comparison: OR = 1.018, 95% CI = 0.791–1.311; dominant model comparison: OR = 1.026, 95% CI = 0.812–1.295). Again, the cancer cases and controls did not significantly differ in the subgroup analyses according to the ethnicity and the source of controls. Intriguingly, the ATM rs664677 polymorphism showed evidence of an association with an increased risk for breast cancer (dominant model comparison: OR = 1.447, 95% CI = 1.203–1.740), but demonstrated a protective role in the development of lung cancer in the meta-analyses stratified by cancer type (dominant model comparison: OR = 0.764, 95% CI = 0.635–0.918; Figure 3b).

**Table 2:** Results of the pooled data analyses for the 9 studied and subgroup analysis for ATM rs664677 and cancer risk.

Variables	n^a^	Case/Controls	CC Versus TT		CT Vs TT		CC/CT Vs TT(dominant)		CC Vs CT/TT (recessive)	
			OR(95% CI)	P^c^	OR(95% CI)	P^c^	OR(95% CI)	P^c^	OR(95% CI)	P^c^
Total	8/(9)^b^	3923/4322, (4470/4862)^b^	1.018(0.791–1.311)^d^	0.003	1.023(0.809–1.292)^d^	0	1.026(0.812–1.295)^d^	0	1.014(0.924–1.113)	0.593
**Cancer types**										
Lung Cancer	3/(4)^b^	1645/1663, (2192/2203)^b^	**0.797(0.644–0.986)**	0.725	**0.756(0.623–0.917)**	0.443	**0.764(0.635–0.918)**	0.448	0.972(0.851–1.111)	0.942
Breast Cancer	3	1456/1746	**1.507(1.213–1.872)**	0.652	**1.422(1.171–1.727)**	0.697	**1.448(1.204–1.741)**	0.696	1.137(0.976–1.324)	0.575
Other cancers	2	822/913	0.808(0.609–1.073)	0.658	0.898(0.730–1.104)	0.104	0.877(0.721–1.068)	0.21	0.865(0.672–1.115)	0.215
**Ethnicities**										
Asian	4	2548/2780	1.119(0.780–1.604)^d^	0.007	1.103(0.772–1.574)^d^	0.005	1.113(0.778–1.591)^d^	0.003	1.033(0.924–1.156)	0.631
European	4/(5)^b^	1375/1542, (1922/2082)^b^	0.909(0.618–1.338)^d^	0.058	0.953(0.679–1.338)^d^	0.01	0.949(0.679–1.327)^d^	0.007	0.974(0.825–1.150)	0.293
**Source of controls**										
Population-based	4/(5)^b^	1806/1955, (2353/2495)^b^	0.927(0.670–1.281)^d^	0.075	0.994(0.764–1.292)^d^	0.055	0.989(0.756–1.293)^d^	0.048	0.963(0.839–1.106)	0.287
Hospital-based	2	822/869	1.225(0.594–2.526)^d^	0.036	1.182(0.567–2.463)^d^	0.029	1.025(0.580–2.504)^d^	0.019	1.051(0.863–1.281)	0.493
Mixed	2	1295/1498	1.012(0.5–2.048)^d^	0.01	0.943(0.424–2.094)^d^	0	0.955(0.439–2.076)^d^	0.001	1.065(0.904–1.255)	0.459

a Number of comparisons;

b recessive model differ from other models in these respects;

c P value of Q-test for heterogeneity test;

d Random-effects model was used when P value for heterogeneity test<0.10; otherwise, fix-effects model was used.

### Test for heterogeneity

There was significant heterogeneity in the homozygote (CC versus TT: P heterogeneity = 0.003), heterozygote (CT versus TT: P heterogeneity<0.001), and dominant model (AA/GA versus GG: P heterogeneity<0.001) comparisons. However, in the recessive model comparison (CC versus CT/TT: P heterogeneity = 0.539), heterogeneity was not found. We evaluated the source of heterogeneity by tumor type, ethnicity, publication year, control source, and sample size. We did not observe any contribution to the substantial heterogeneity.

### Sensitivity analyses

Sensitivity analyses were conducted to ascertain the primary origin of the heterogeneity. Four independent studies by Sang-Ah Lee [15], Stefano Landi [18], Kyoung-Mu Lee [20], and Sandra Angele [13] affected the heterogeneity. The heterogeneity was effectively decreased or removed by the exclusion of these four studies (CC/CT versus TT: P heterogeneity = 0.601). Furthermore, no single study changed the pooled ORs qualitatively, suggesting that the results of this meta-analysis were stable.

### Publication bias

Begger's funnel plot and Egger's test were used to identify the potential publication biases of the literature, the shapes of the funnel plots appeared to be symmetrical (Figure 3), suggesting that there was no obvious publication bias. Egger's test was used to provide further statistical evidence; similarly, the results showed no significant publication bias in this meta-analysis (t = −0.02, P = 0.984 for cc vs. tt)

## Discussion

In response to DNA damage, the dormant kinase and sensors of ATM are rapidly activated, and various downstream substrates of ATM, which compose an ever-expanding network, are phosphorylated. Some of the downstream substrates are key factors in the regulation of cell-cycle arrest, DNA repair, and apoptosis. Given the important roles of ATM in response to DNA damage, inherited variability in this gene could directly or indirectly contribute to susceptibility to cancer [12].

Several studies have reported the associations between several genetic variants of ATM and risk of cancer, for instance, rs1800057 (P1054R), rs1801516 (D1853N) and rs1800054 (S49C) [25]–[28]. Some of these SNPs involved in the nonsynonymous variants which caused the amino acid alteration and might have a physiologic effect on cancer development. Here, we focused on the association between a synonymous SNP (rs664677) and cancer risk. ATM IVS 22–77 T>C (rs664677) is located in the noncoding region. One of the possible mechanisms for IVS 22–77 T>C in the ATM gene seemed to be mediated by affecting RNA splicing [29]. The other possible mechanism might be the influences of mRNA stability. However, the actual mechanism of IVS 22–77 T>C in the ATM gene remains uncertain.

The present meta-analysis of 9 studies, including 4470 cases and 4862 controls, provided evidence that there is no association between cancer and the ATM rs664677 polymorphism. The results from a recent meta-analysis investigation of the association between the ATM D1853N polymorphism and breast cancer risk presented evidence consistent with our results [30]. Moreover, in subgroup studies by source of controls and ethnicity, no significant associations were found in any genetic models. However, the rs664677 polymorphism in the Asian and European populations was observed to have an inverse association with cancer risk in all genetic models, although without any significance. Considering that the ATM polymorphism presents with different frequencies in different populations, analysis of the data respectively from the various ethnic groups might eliminate some bias. And this discrepancy we observed may be due to the difference in the source of the controls. The source of controls of the initial studies might have a weak effect on the results in our analysis. However, we thought the population-based controls were more representative of the general population. Thus, in genetic association studies, the selection of controls and matching status should be carefully considered. If we use the population-based controls, we can obtain a higher reliability.

In the subgroup analysis according to cancer type, the odds ratio for CC homozygosity versus heterozygosity plus TT homozygosity was decreased for lung cancer, but this was not statistically significant (0.972; 95% CI = 0.851–1.111). In other models, however, the OR was significantly decreased. In contrast to lung cancer, where the ATM rs664677 C allele is protective, in breast cancer, it seems to be associated with an elevated risk. Such differences have been previously reported; for example, the CHEK2 I157T variant with the rare allele conferred an elevated breast cancer risk but a protective effect on lung cancer [31]. Interestingly, we observed the association exhibit a disaccord in breast and lung cancer risks, which could be caused by the following two reasons: one might be that non-genetic factors are likely to have entirely different mechanisms that affect tumorigenesis in concert with genotype. For instance, gene-environment interactions might modulate cancer risk. The other possible reason is that ATM utilizes diversity mechanisms that regulate cell proliferation or apoptosis in different cancer cells.

Recent genome-wide association studies (GWAS) have reported several SNP to be associated with breast or lung cancer, including rs1219648, rs1092913, rs2736100, rs16969968, rs8034191, and rs402710 et al [32]–[41]. While no GWAS data reported the association of rs664677 with breast or lung cancer to date. As we known, in GWAS, four key points (models of the allelic architecture of common diseases, sample size, map density and sample-collection biases) need to be taken into account in order to optimize the cost efficiency of identifying genuine disease-susceptibility loci [42]. Due to the strict criteria, some low-risk alleles might be overlooked in spite of their potential importance in disease risk.

A literature reviewed that eight hallmarks constitute an organizing principle for rationalizing the complexities of neoplastic disease. They include sustaining proliferative signaling, evading growth suppressors, resisting cell death, enabling replicative immortality, inducing angiogenesis, activating invasion and metastasis, reprogramming energy metabolism and evading immune destruction [43]. Studies with ataxia telangiectasia (A–T) cells and ATM-deficient mice have shown that ATM is a key regulator of the multiple signaling cascades that respond to DNA strand breaks induced by damaging agents or by normal processes, such as meiotic or V(D)J recombination. These responses involve the activation of cell cycle checkpoints, DNA repair and apoptosis [7]. Considering the ATM multifunction, it might affect several pathways in the hallmarks and therefore involve diverse mechanisms in different cancer types. To date, the pathway through which the ATM polymorphism acts is unclear. Further research is necessary.

Heterogeneity is a potential problem that might affect the interpretation of the results. Significance between the meta-analysis heterogeneity existed in almost all comparisons, except the recessive model. We detected the source of heterogeneity by tumor type, ethnicity, publication year, control source, and sample size. However, there was no evidence to determine which of them contributed to the substantial heterogeneity. One possibility involves differences in the matching status. However, we cannot confirm this possibility because no detailed information was provided. Heterogeneity could also have resulted from the fact that each study used a different approach to select participants. However, it seems unlikely that the selection procedure would affect the genotype at the locus. Thus, we do not have a clear explanation for the statistical heterogeneity that was present for the SNP. The leave-one-out sensitivity analysis would not have materially altered the results of this pooled analysis, indicating that our results were robust. The publication bias for the association between this polymorphism and cancer risk was not observed.

### Limitations

Several potential limitations of the present meta-analysis should be taken into consideration. First, although the funnel plot and Egger's test showed no publication bias and although an exhaustive literature search was done, it is likely that some publications and unpublished data were overlooked. Selection bias for the meta-analysis might have occurred. Secondly, in the subgroup analysis by cancer type, the number of studies and subjects analyzed for rs664677 was small, and the statistical power was so low that caution should be taken in interpreting these results. A further investigation with much larger sample sizes is needed. Thirdly, our results were based on unadjusted estimates due to the absence of available information. A more precise analysis would be detected if more detailed individual data were available, such as age, sex, and exposure. Despite its limitations, our meta-analysis also had some advantages. There was no evidence for heterogeneity in a recessive model among the studies of this SNP. We found a paradoxical role for the ATM rs664677 polymorphism contributing to both cancer suppressing and promoting effects.

In summary, this meta-analysis convincingly demonstrated that the ATM rs664677 polymorphism is not associated with cancer risk. A moderately protective effect was observed with the lung cancer risk. In contrast, we observed an elevated risk of breast cancer susceptibility. In conclusion, well-designed, unbiased studies should be done to gain a more comprehensive understanding of the association between the ATM gene and cancer risk.
